# Suppressing the Mosquito

**Published:** 1881-05

**Authors:** 


Suppressing the Mosquito.—Professor Fontaine gives some hints for abating the mosquito pest which is sure to come with the advent of sunny days. He says mosquitoes require water for the deposit of their eggs and the rearing of their larvæ or wiggle-tails. Therefore all cisterns should be made close and covered with close, woven brass wire setting to prevent their laying in them. No old tubs, barrels or receptacles of water, ought to be permitted, and no stagnant pools left undrained within a mile of any dwelling. Then they can be killed by the cheapest and most abundant of all alkalies, common lime. Therefore this ought to be poured into every cesspool and spring. A pound of strong lime to every 100 gallons of stagnant water is sufficient. But even a pound to 1,000 gallons of a cistern of drinking water will kill them, although it will probably give the water an unpleasant flavor and make it too " hard " for most domestic tastes.

New Empress of Russia.—The Princess Dagmar, as the empress is still called in the land of her birth, grew up with her sister Alexandra, now the Princess of Wales, at the Danish court with very modest surroundings. The queen was an excellent mother, and sought to develop in her daughters the woman in preference to the princess. It used to be said at the capital that the princesses were made to help in making their own dresses, and that the furniture in their common bed-room was covered with inexpensive calico.

### CUNNING AND PLAYFUL.

The morality of an elephant is even a more curious study than his sagacity. A gentleman in Rangoon bought three young elephants to send to England. They are said to be very tame, cunning and playful. They know it is wrong to steal paddy (unhusked rice), and, though they know where it is kept, they will not touch it themselves, but when the boys come to see them they will come up and coil their little trunks around a boy's arm and pull him along to the stable and up to the paddy bag, and make a cat's-paw of the boy's hand until he takes up a handful of paddy. Then he lets go the arm, and turns up the end of the trunk, opens it like a cup, and most coaxingly invites the boy to drop in the paddy. If the boy puts it back into the bag, he instantly seizes his arm again and makes him try once more, until he gets the paddy in his trunk; then he doubles his trunk under, opens his mouth, and blows the paddy out into his mouth and scampers off, feeling as jolly as a boy does when he thinks he has done a funny thing.

---

The bush of Australia is so overfed by the multiplying of wild horses that they have to be shot down in common with rabbits and kangaroos. In one district an Arab stallion got away some thirty years ago, and was never recaptured. He was a chestnut, and took a couple of thoroughbred colts with him, and it has been remarked that a large proportion of the wild horses of the district are of his color. Horses believed to be very old are occasionally seen far away in distant ranges. One man has shot 3,000 horses in two years.

---

A man doesn't necessarily imperil his immortal soul by eating with his knife, but he stands a fair chance of slitting the top of his head off, besides leaving the impression that he is not posted on table manners.—*Steubenville Herald.*

---

### "LATITUDE UNKNOWN."

Like lonely sailors on a foreign sea,
Without a compass and without a chart,
Unhelped by all their lore of seaman's art,
Souls drift along in the vast mystery
Of Love's companionship. There cannot be
A solitude so pathless as a heart.
No undiscovered isles lie so apart
From him who seeks, as lie the thoughts that we
Forever yearn to read behind dear eyes—
The dear eyes that we love, and love to kiss.
Ah, well! But one thing matters to our bliss.
So long as Love's sun goes not down, all skies
Are clear: all shores are friendly: treasure lies
On all: we shall not one sweet harbor miss.

—*H. H., Scribner for June.*

A CLINICAL professor, surrounded by medical students, is at the bedside of a patient. "What is your profession?" "A musician, sir." The professor, turning to his pupils: "As you see, gentlemen, this poor man has an affection of the lungs. Here is an opportunity of proving to you the truth of all that I have told you so frequently in the lecture-room, viz., that the fatigue and the effort to the respiratory organs in blowing a musical instrument are frequently the cause of the illness from which this man is suffering." Then, again addressing the sick person: "What instrument do you play?" "The cymbals and the big drum, sir?" Tableau!

MEDICAL USES OF EGGS.—For burns or scalds nothing is more soothing than the white of an egg, which may be poured over the wound. It is softer as a varnish for a burn than collodion, and being always at hand can be applied immediately. It is also more cooling than the "sweet oil and cotton" which was formerly supposed to be the surest application to allay the smarting pain. It is the contact with air which gives the extreme discomfort experienced from ordinary accidents of this kind; and anything which excludes the air and prevents inflammation is the thing to be at once applied.

THE old custom was to call young women Miss and older women Mrs., the age of 30 being about the dividing line, regardless of whether they were married or not. Elizabeth A. Kingsley writes to the *Women's Journal* that the usage was right and ought to be revived. "It is annoying," she says, "to be introduced to Mrs. Brown, a silly, superficial creature yet in her teens, and the next moment to be presented to Miss Williams, who at a glance we perceive to be an intellectual, noble, broad-souled woman of thirty-five or forty, worth more than a dozen like Mrs. Brown."

TABLEAU VIVANT.—Bridegroom (to his little sister-in-law at the breakfast)—"Well, Julie, you've got a new brother, now—" Julie (enfant terrible)—"Yes; and ma said the other day to pa, she didn't think he was much account, on'y it looked like Lottie's last chance!" (Great clatter of knives, forks and spoons).—*London Punch*.

A CINCINNATI paper avers that "women dress too hastily." This clearly is "sarkasm," or else the editor never sat for two mortal hours in a hired cutter, waiting for Angelina to "be down in a moment."—*Catskill Recorder*.

"I SHOULD blush to simper," is the latest slang.

### SANITARY DEPARTMENT OF "HALL'S JOURNAL OF HEALTH."

We are daily receiving requests from our readers to purchase for them "HEALTH FOODS," "FOOD MEDICINES," PURE BAKING POWDERS, and various MEDICINES that our experience has taught us the value of; also standard books; and last but not least, many families rely on us to procure for them PURE BOVINE VACCINE MATTER, which is the only vaccine matter that is always safe. These commissions already keep one person employed much of the time. We have therefore determined to make this a branch of our regular business, and announce ourselves ready to fill orders for GLUTEN of WHEAT, GLUTEN COFFEE, COOKED FOODS, as CRUSHED WHEAT, BARLEY, OATS, CORN and RYE, also the various preparations of MALT, and for books, medicines, vaccine matter and any other like articles that can be found in New York market.

E. H. GIBBS & CO.

---

IT is related of a certain clergyman, who was noted for his long sermons with many divisions, that one day, when he was advancing among the teens, he reached at length a kind of resting place in his discourse, when pausing to take breath and asking the question, "And what shall I say more?" a voice from the congregation earnestly responded, "Say 'amen!'"

LOVE AND THRIFT.—A New Hampshire farmer recently agreed to sell his farm for $2,000, but when the day came he told the expectant purchaser that his wife was in hysterics about the trade, and he "guessed he'd have to back out." The purchaser complained, and finally asked how much more would induce him to sell. "Well," replied the thrifty son of the Granite State, "give me $250 more, and we'll let her cry."—*Boston Transcript.*

"You have seen Mrs. Langtry?" asked one of her London admirers, addressing himself to an American tourist. "Oh, yes," was the answer. "And what did you think of her?" "Well," drawled the American, "I have nothing agin her that I know of. I was thinking all the time that it was her dress maker who was to blame."

"WHY don't you put the tooth-picks on the table?" asked a guest at a Galveston hotel, after he had finished his dinner. "Because after you used one yesterday you didn't put it back in the saucer," responded the new waiter.—*Galveston News.*

TAKEN on the spot—The measles.

A LAND league—Three miles.

### LIFE INSURANCE WITHIN THE REACH OF ALL.

INSURING one's life, which most people deem a duty, has hitherto been a luxury too costly for people of moderate means. Life insurance companies, in order to accumulate their enormous surplus funds, in many cases amounting to millions of dollars, have fixed their rates of premiums at figures which, we repeat, are prohibitory.

The extravagance of what we may properly call this old system of insurance has driven great numbers of persons to the adoption of a newer and better system of life insurance, within the reach of those to whom it is of the greatest use, to wit, persons of moderate means. This system is strictly mutual, and therefore the safest of any ; since, after providing for the ordinary expenses of economical management, members are only taxed the small sums required from time to time to make up the losses by death ; thus members are not taxed three or four times as much as is necessary in order to establish an enormous surplus fund in which they have no interest beyond the amount of their insurance. We believe in the new plan of life insurance, and have shown our faith by taking policies of insurance in two associations. We will forward all necessary printed information to any reader of the JOURNAL who will take the trouble to write to us on the subject.

**KNIGHT BROTHERS,**

SOLICITORS OF

UNITED STATES AND FOREIGN

**PATENTS,**

**Counsellors in Patent Causes**

AND

**MECHANICAL EXPERTS,**

AGENCIES AT PRINCIPAL FOREIGN CAPITALS.

**No. 633 F Street, N. W., Washington, D. C.;**
**75 West Fourth Street, Cincinnati, Ohio;**
**317 Olive Street, St. Louis, Mo.**

**SEND FOR BOOK OF INSTRUCTIONS.**

### DR. HALL'S PORTABLE SPIROMETER.

NO PHYSICIAN questions the value of the spirometer for exercising and expanding the lungs, and for measuring their capacity. The great difficulty has been to find an instrument that can be compressed into a small compass when not in use, so that the physician or patient may conveniently carry it from place to place as circumstances require. Another obstacle to the more general use of this truly valuable instrument has been its price. The unwieldy spirometer of a few years ago cost from $10 to $20. Dr. Hall's Portable Spirometer sells for $7.50, and will be sent free to any address on receipt of the price by the editor of this journal.

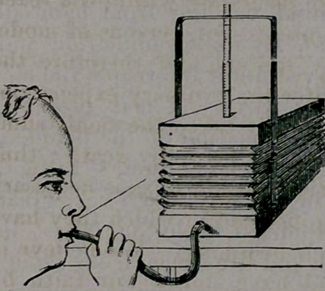

It is 6 by 12 inches, and when closed it stands only 3 inches high, and weighs less than 3 pounds.

This cut represents Dr. Hall's Portable Spirometer, partly inflated, in order to show the manner of using it. When not in use it can be closed together so as to occupy the small space above stated. The scale and guide are hinged, so that they close down on the top of the spirometer, and occupy but little space. The top is of polished black walnut. The brass-work is plated with silver or nickel.

### HOW TO USE THE SPIROMETER.

The instrument should be used only in a perfectly ventilated room, which is entirely free from dust, or else in the open air, away from the dust and smoke and bad odors of the streets of a city. When it is remembered that the capacity of the lungs of a man of medium size is 150 to 250 cubic inches, and that in the act of breathing naturally, or rather as some have acquired the habit of breathing—which is far from natural—the lungs take in only from 20 to 40 cubic inches of air at each inspiration, leaving from 130 to 230 cubic inches of air cells almost always in a dormant condition, the importance of regular and systematic exercise for the lungs will be appreciated. But, like all good things, this sort of lung exercise must be indulged in moderation. Draw a full breath and then blow through the rubber tube into the spirometer; at the same time watch the figures on the scale as it rises. The figures seen on the scale as it passes through the top of the guide indicate the number of cubic inches of air blown into the spirometer.

It is now very generally conceded that mankind start quite evenly on the journey of life, in spite of supposed hereditary tendencies to pulmonary consumption, and that with proper care the average term of life is not shortened by these hereditary tendencies. This being the case, it is far better to enjoy health and long life through means which the naturally robust are not compelled to resort to, than to believe in or lament over the inheritance of ills that do not exist. We believe for all persons who are confined much of the time in doors, particularly in cases where there is a tendency to weak lungs, the regular use of the spirometer in the open air is highly beneficial. Its use just before bedtime is said to induce sleep, by drawing blood from the brain to the lungs.

### CONSTIPATION.

CONSTIPATION becomes alarmingly frequent as people acquire the means of living luxuriously without regular manual labor. It is still more frequent among persons who become so through ignorance of the penalties that follow a want of attention to the regular demands of nature.

When fecal matter is retained in the rectum more than twenty-four hours, it commences its destructive work, notably in two ways. *First*, It is absorbed into the system, and acts as a blood poison. One of the worst results from blood poisoning from constipation is to cause disease of the mind, varying from habitual moroseness to actual insanity. *Second*, By its accumulation and impacting in the lower portion of the rectum it interrupts the circulation of the blood in the adjoining parts, and may produce, from this cause alone, piles, fistula, inflammation of the bladder, disease of the prostate gland, or cancer of the rectum. In fact, most of these diseases are developed in this way. To a very great extent we hold our health and happiness, even our lives, in our own hands. If we sacrifice health and life through our ignorance, or indifference, we pay the penalty through suffering, more severe because more prolonged than instant destruction. From whatever cause, there is, unfortunately, great ignorance in regard to the laws of health among a class of people who are otherwise intelligent. How many parents are there who look carefully to the habits of their children, upon which all that will make life desirable for them so much depends? Cathartics and clysters should not be used in constipation. They increase the trouble.

That which has, perhaps, met with most general favor relates to the diet. Certain foods have been regarded as specifics for this complaint, and particularly "Graham bread." This often induces activity of the bowels, but it ruins digestion like other cathartics, in a manner which we have heretofore fully explained in the pages of this journal. Kneading the bowels has sometimes afforded relief, but it affords no permanent benefit, as a rule. We have, however, found a remedy for constipation and for piles which is always safe, and is attended with no inconvenience or discomfort. We have prescribed it in hundreds of cases. It has usually afforded prompt relief, and many patients who have suffered for years believe that they have been permanently cured of constipation by its use. This remedy is "Nelaton's Suppository." It is prepared and sold by Hall & Ruckel, wholesale druggists, 218 Greenwich street, New York, at 50 cents and **$1.00 a box, and is sent free by mail on receipt of price.**

